# Combined antibacterial effect of essential oils from three most commonly used Ethiopian traditional medicinal plants on multidrug resistant bacteria

**DOI:** 10.1186/s12906-019-2429-4

**Published:** 2019-01-18

**Authors:** Eshetu Gadisa, Gebru Weldearegay, Kassu Desta, Getahun Tsegaye, Sityehu Hailu, Kefiyelewu Jote, Abera Takele

**Affiliations:** 1 Kotebe Metropolitan University, P.O. Box 3268, Addis Ababa, Ethiopia; 2 Department of Medical Laboratory Science, CHS, AAU, Addis Ababa, Ethiopia; 3 Department of Human Physiology, CHS, AAU, Addis Ababa, Ethiopia; 4 CHS, Madda Walabu University, Addis Ababa, Ethiopia; 5 Salale University, Fiche Campus, Addis Ababa, Ethiopia

**Keywords:** *Blepharis cuspidata*, *Boswellia ogadensis*, *Thymus schimperi*, MRSA

## Abstract

**Background:**

An alarm increase the rate of emerging and re-emerging of multidrug resistant bacteria have been caused great public health concern in the worldwide. They have been resisting for most or majority of currently available and affordable antibiotics and imposed socioeconomic catastrophe at global scale. As a result, there is utmost important to discover new or modify currently available antibiotics. The aim of this study was to evaluate combined antibacterial effect of essential oils obtained from *Blepharis cuspidata, Boswellia ogadensis* and *Thymus schimper* against multidrug resistance (MDR) *Escherichia coli, Klebsiella pneumoniae* and *Methicillin resistant S. aureus.*

**Methods:**

Essential oil (EO) was extracted from the aerial part of *B. cuspidata, B.ogadensis* and *T. schimper* by steam distillation and stored in brown bottles at 4 °C. There were mixed in 1:1 ratio and adsorbed to disc and placed on MHA and measured their minimum inhibitory zone seeded with *E. coli, K. pneumoniae* and *MRAS* after 18-24 H*. minimum* inhibitory concentration (MIC) and minimum bactericidal concentration (MBC) were measured by broth micro-dilution method. The interaction between EOs was determined by fractional inhibitory concentration index.

**Results:**

The antibacterial potential of mixed oil depends on the doses and type of the EOs and bacteria species. The combined EOs of *B.cuspidata* and *T.schimperi* had inhibition zone (39 mm), its MIC and MBC value was 0.39 μl/ml against MRSA. It had inhibition zone (28-35 mm), MIC value 0.39–6.25 μl/ml and MBC (0.78–12.5 μl/ml) against MDR *E. coli* and *K. pneumoniae*. Whereas, combined effects of *B. cuspidata* and *B. ogadensis* had MIC values ranges from 0.78–6.25 μl/ml for *E.coli* and *K. pneumoniae* and 1.56 μl/ml for MRSA. There was strong synergistic effect between the combination of *B.cuspidata* and *T.schimperi.* This study revealed that gram negative bacteria were slightly less susceptible than gram positive.

**Conclusions:**

This in vitro study of combined EOs has significant antibacterial effect than using each of them and even it was more potent antibacterial effect on MDR as compare to modern antibiotics. Hence, it can be applied to a pharmaceutical composition as modulator or adjuvant or precursor for synthesis of new antibiotic in future activities*.*

## Background

An alarm increase the rate of emerging and re-emerging of multidrug resistant bacteria have been caused serious difficulties in the treatment and continued to be clinical and public health concern in the worldwide [1, 2]. Spreading of methicillin-resistant *S. aurues* [3, 4] and ESBL producing *E. coli* and *K. pneumoniae* become difficult to treat [5, 6]. These bacteria have been continuing to develop resistance for most currently available antibacterial drugs by either mutation or exchange of genetic information [7, 8]. Many resistance mechanisms that emerge and spread in bacteria population are widespread. Recently, discovered factors with major implication for the emergence, dissemination and maintenance of resistance include multidrug efflux, hyper-mutability and plasmid addiction [2, 3, 9]. Such factors have been compromised all or majority of the drugs belonging to a given therapeutic [7, 8]. As a result, it has been initiated for searching a new, better and affordable antibiotic derived from medicinal plant as alternatives or complementary treatment for drug resistance microbial including bacteria [10, 11].

In developing countries, the majority of the population still can’t afford to purchase modern pharmaceutical drugs and continued to use indigenous traditional medicinal plants [12]. Of which, tropical plants are the most valuable source of new bioactive due to their biodiversity coupled with the chemical diversity found within each species [13, 14]. However, higher plants in general and endemic medicinal plants of our country in particular haven’t been screened from the viewpoint of bioactive for phytochemical and pharmacological utilization from a wider perspective [14]. Hence, there is a need to carry out proper research in order to investigate the efficacy and safety of herbal remedies.

With the dearth of novel antibiotics, traditional healers’ extracted Eos from TMP used for treatment of different illness [15]. The chemical composition of essential oils isolated by steam distillation contain varies bioactive compound that exhibited remarkable bacteriostatic and bactericidal activities [14, 16]. It is likely that their mode of action involved several targets in the bacterial cell. Some of them acted on partition in the lipids of the cell membrane resulted in leakage of cell contents [16], inhibited cell cycle (S-phase), inhibited protein synthesis and DNA replication [17, 18]. Therefore, they are purposed as promising antibiotic to overcome the multidrug resistance bacteria.

The combined effects of modern antibiotics (ciprofloxacin, ceftazidime and tetracycline) and six phytochemicals (protocatechuic acid, gallic acid, ellagic acid, rutin, berberine and myricetin) showed that it had inhibited the growth of *P. aeruginosa*. This combination of modern antibiotic and natural compounds were revealed more antibacterial effect than single compound [15, 19]. Studies done on *MRSA* and *ESBL* produced *Enterobacteriaceae* showed that EOs was radically reduced their growth [20, 21]. On the other hands, study done on MDR bacteria showed that its growth was inhibited by extracts from clove, jambolan, pomegranate and thyme [22]. Likewise, in-vitro interaction between the tested antimicrobial and eleven EOs showed promising effect against drug resistance *S. aureus, E. coli, K. pneumoniae, P. aeruginosa* and clinically isolated strains [22–24].

The combined plant extract exhibited more antibacterial effect on the MDR bacteria than any of the individual plant extracts [23]. As a result of different phytochemicals such as coumarins, flavonoids, phenolic, alkaloids saponins, tannins, terpenoids, quinones, anthraquinones, cardiac glycosides and others are found in each plant in different concentration [24, 25]. Many studied showed that combination of selected phytochemicals and antibiotics was resulted in foliate biosynthesis inhibitors, DNA/protein synthesis inhibitors and cell permeability/cell wall inhibitors [10, 18, 19]. Many studies showed that Eos also inhibit macromolecules (DNA, RNA, protein and polysaccharide) synthesis in pathogen bacteria [15, 24]. Synergistic effects of essential oils can provide effective therapy against multidrug resistant bacteria [23]. These synergistic combinations represent a largely untapped source of new pharmaceutical products with novel and multiple mechanisms of action that could overcome pathogenic microbial resistant [19, 22–25]. With this notion, we purposed to evaluate combined antibacterial effect of essential oils from *B. cuspidata, B. ogadensis* and *T. schimper* against MDR bacteria. This study could serve as a baseline data to investigate new bioactive from essential oil and find out the scientific rationale for the combined effects of untapped traditional medicinal plants used by different societies.

## Methods

In vitro experimental study was employed to evaluate combined antibacterial effect of essential oils obtained from *B. cuspidata, B. ogadensis* and *T. schimperi* against clinical isolated MDR gram negative (*E.coli* and *K. pneumoniae*) and *MRSA* and their reference strains.

### Medicinal plants selection criteria

In this study, plant selection was on the basis of knowledge of herbalist lived in Bale zone. Those herbalists were used for treating various skin diseases, urinary tract infection sexual transmitted infection, hypertension, tumorcidal, sexual impotence and others. Dawe Kechen and Dawe Serare are found in Bale zone, south east Ethiopia. They are the most remote area with no infrastructure (transport and power supplies). Until this field work, there is no hospital; even one health center with no full functioning the health activity in each district. As a result, the community imposed to use medicinal plants for treatment of various diseases. For instance, *V. schimper* (Qorsa finchaanii), *C. myricoides* (Handaraafa) and *Z. scabra* was used to treat cancer, tumor, urinary tract infection and gonorrhea. Whereas, *B. cuspidate* (Qoree waraantii), *B. ogadensis* and *B. edulis* (Suree Lukkuu) used for treatment of kidney, liver cirrhosis, hepatitis, skin diseases, cancer and diabetes.

### Plant collection and preparation

Essential oils obtained from aerial parts of *T. schimper, B. cuspidate* and *B. ogadensis* were evaluated for their antibacterial effect on multidrug resistant bacteria. *T. schimper* was collected from Dawe Kechen. *B.cuspidate* and *B. ogadensis* were collected from Dawe Serare. Authentication of each plant sample was carried out in the Department of Biology, Faculty of Natural and Computational Science, Addis Ababa University by Dr. Melaku Wondafrash. Those identified plant samples were deposited at the National Herbarium with voucher number *Thymus schimperi* (E-25/07), *Blepharis cuspidate* (E-11/07) and *Boswellia ogadensis* (E-09/07).

### Extraction of essential oils

Health and well grown fresh leaves of each plant was collected and cut into small pieces. Plant materials were washed thoroughly under running tap water. Then, it’s subjected to steam distillation using AMIO-37/04 model for 4 h. Essential oils were extracting from aerial parts of *T.schimperi*, *B. cuspidate* and *B. ogadensis* as guideline described by WHO on quality of herbal medicine. The purified essential oils were stored in brown colored bottle vials at 4 °C until used [9, 11, 26, 27].

### Culture media

Nutrient agar, MacConkey, Muller Hinton agar (MHA), Muller Hinton Broth (MHB), blood agar (BA), manitol salt agar (MSA), chocolate agar and biochemical reagents were obtained from Department of Medical Microbiology, Immunology and Parasitology, CHS, AAU and Tikur Anbessa Specialized Hospital (TASH), Bacteriology Unit.

### Test organisms

The reference strains of *S. aureus* (ATCC25923), *E.coli* (ATCC25922), *K. pneumoniae* (ATCC700603) were obtained from TASH and Ethiopian Public Health Institution and their multidrug resistant strains isolated from different samples of patient’s attending TASH according to CLSI guideline [26].

### Modern antibiotics

Modern antibiotics such as tetracycline (30 μg), ciprofloxacin (5 μg), gentamycin (10 μg), cephalotaxon (30 μg), chloramphenicol (30 μg), cefotaxime (5 μg), ceftazidime (10 μg), ceftriaxone (30 μg), amikacin (30 μg), cefuroxime (5 μg), ceftriaxone (30 μg), cefoxitin (30 μg), cloxacillin (5 μg), augmentin (30 μg) were used for testing MDR bacteria according to CLSI guideline [26].

### Screening for multidrug resistant bacteria

Multidrug resistance gram negative and *S. aureus* were isolated from clinical specimen such as CSF, urine, wound and blood. Triplicate of each *MRSA* and MDR gram negative bacteria were isolated from clinical specimen. All bacterial cultures were first grown on 5% blood agar plates at 37 °C for 18 to 24 h prior to inoculation onto the MHA. Few colonies (3 to 5) of similar morphology of the respective bacteria were transferred with a sterile inoculating loop to a liquid medium until adequate growth of turbidity with McFarland of 0.5. Then the bacterial suspension was streaked on MHA plates using a sterile swab in such a way as to ensure thorough coverage of the plates and a uniform thick lawn of growth following incubation. The susceptibilities of clinical isolates were tested by using the MHA contains a range of antimicrobial agents. Dilutions of overnight broth cultures were inoculated onto antibiotic containing plates to yield final inoculums of approximately 10^6^ CFU per spot according to CLSI for *MRSA* and *E.coli* and *K. pneumoniae* [26, 28].

#### Screening for gram negative

Selected multidrug resistant gram negative such as *K. pneumoniae* and *E. coli* were screened for their resistant for more than two different classes of antibiotics following disk diffusion method as CLSI guideline and WHO recommendation [26].

#### Screening for methicillin resistance *Staphylococcus aureus*

In this study, cefoxitin was used as marker of *mecA /mecC* mediated by methicillin resistant *S. aureus* and drug of choice for disk diffusion method as recommended by CLSI guideline [26]. This strain was selected based on antibiotic profile mentioned (Table 1).

**Table 1:** Antibiotics resistant profile of multidrug resistant bacteria at AAU, 2018

Modern drugs	Drug susceptibility test for multidrug bacteria strains								
	S.1	S.2	S.3	E.1	E.2	E.3	K1	K2	K3
Ampicillin	R	R	R	N	R	R	N	I	R
Augmentin	R	I	S	R	R	R	R	R	R
Chloramphenicol	R	R	R	S	S	S	R	R	R
Erythromycin	R	R	S	R	S	R	I	N	S
Gentamycin	N	N	N	R	R	I	R	S	N
Ceftriaxone	N	N	N	R	R	R	R	R	R
Ceftazidime	I	R	R	N	N	N	R	R	R
Amoxicillin	R	R	R	R	R	R	R	R	R
Cefiroxime	R	R	R	S	R	R	N	R	R
Cefoxitin	R	R	R	N	N	N	R	R	R
Ciprofloxacin	S	S	S	S	S	S	S	S	S
Norfloxacin	N	N	N	S	S	S	S	S	S
Amikacin	R	I	R	R	R	I	R	R	S

*S S. aureus, E E. coli, K K. pneumoniae, N* Not done, *R* Resistance (≤ 16 mm IZ), *S* Sensitive (≥21 mm IZ) I = Intermediate (17-20 mm IZ) according to CLSI guideline [26]

### Determination of MIC and MBC values

After preliminary screening of essential oils, those revealed potent antimicrobial effect were further tested to determine MIC and MBC for MDR gram negative bacteria *(K. pneumoniae* and *E. coli*) and MRSA. It was determined by MHB broth micro-dilution method. Each 96-well micro-titer plate was liquated with 50 μL of MHB; 10^th^well (sterility control) was added with 100 μL of MHB. And the 9^th^well (growth control) was added with MHB with 5% DMSO. 50 μL of essential oils initially dissolved in 5% DMSO was added into the first well. A serial 2-fold dilution was performed by transferring 50 μL of the suspension to the subsequent wells up till the 8^th^well; this procedure was performed by modifying Wiegand protocol. 0.5 McFarland broth inoculum was diluted in the ratio of 1:100 and added into 1st-8^th^well in achieving the final inoculums size at 5 × 10^5^ CFU per ml [27].

Bacterial cell viability and MIC values were determined by observing the turbidity. The lowest concentrations of essential oils with clear suspension were considered as the MIC values. The lowest concentrations of essential oils in the post-incubation suspensions which did not harbor any bacterial growth upon spotting on MHA after overnight incubation at 37 °C were considered as the MBC values. Test was performed in triplicates alongside antibiotics ciprofloxacin (5 μg) for gram negative and cefoxitin (30 μg) for *MRSA* as positive control respectively [5, 9, 26, 27].

### Fractional inhibitory concentration index

In vitro drug interaction was determined by the checkerboard method as described elsewhere and the results were analyzed with the FIC index. Growth control wells containing medium were included in each plate. Each test was performed in triplicate. The concentration of antibiotics needed to inhibit growth was recorded. The following formula was used to calculate FIC: MIC of drug in combination FIC/ MIC of drug alone = The FIC index (∑FIC) calculated as the sum of each FIC, was interpreted as follows: synergy is defined as a FIC index of ≤0.5. Antagonism is defined as a FIC index of ≥2. An indifferent/additive effect is defined as a FIC index of 0.5 < X < 2 or a micro dilution decrease of 1 dilution in the MIC of one or the other drug or no change in the MIC of either of the drugs [24].

### Statistical analysis

Statistical data were reading values of inhibition zones (in diameter) and concentration values (MIC &MBC) analyzed using SPSS, version 21 according to CLSI. Each experiment values are expressed as mean ± S.D. Statistical significance was determined by student’s t-test. Values with *p* < 0.05 were considered significant.

## Results

### Antibacterial effect of modern antibiotics

The present study revealed that tested multidrug resistant strains isolated from clinical samples were resisted for the majority of currently available and affordable antibiotics in developing countries. The clinical isolates, those referred to as methicillin resistant *S.aureus* (MRSA) were selected on the basis of their resistance to cefoxitin in a disk diffusion assay. This gram positive bacterium was resisted not only cefoxitin but also resisted for amikacin, amoxicine, ampicillin and cefotaxime (Table 1). On the other hand, gram negative bacteria*(E. coli* and *K. pneumoniae*) was unpredictably resisted for third generation cephalosporine and most commonly used penicillin classes (Table 1). Such resistant strains had developed genes or gene products that enable them to resist for tested antibiotic. Unless and otherwise, new drugs or modify currently available antibiotic will be revolutionized, emerged multidrug resistant bacteria impose potentially large health and socioeconomic burden on societies and worries future provision of health care services.

### Antibacterial effect of essential oils

This study revealed that essential oils extracting from *B. cuspidata, B. ogadensis* and *T. schimper* were demonstrated antibacterial effect against tested bacteria on their 10 μl/disc (Table 2). They had appreciable antibacterial effect not only against reference strains of *S. aureus*(ATCC25923), *E. coli* (ATCC25922) and *K. pneumoniae* (ATCC70603) but also on multidrug resistant strains isolated from clinical specimens. Their effectiveness varied with concentration, type of the essential oils and the type of bacteria species. Overall, all essential oils had overriding antibacterial effect against gram positive and gram negative bacteria.

**Table 2:** Inhibition zone (mm) of essential oils against MDR bacteria at AAU, 2018

Medicinal plant	Con (μl/ml)	μl/disc	Gram positive		Gram negative			
			MSSA	MRSA	*E. coli*		*K. pneumoniae*	
					R	S	R	S
*T.schimper*	100	10	16 ± 0.9	16 ± 0.4	11 ± 0.4	10 ± 0.7	17 ± 0.4	17 ± 1.2
	100	20	21 ± 0.6^*^	21 ± 0.3	17 ± 0.4	13 ± 0.1	24 ± 0.2^#^	24 ± 1.0
*B. cuspidata*	100	10	19 ± 0.3	19 ± 0.2	15 ± 1.3	11 ± 0.3	19 ± 0.3	18 ± 0.2
	100	20	22 ± 0.6^*^	25 ± 0.1	19 ± 0.8	15 ± 0.1	23 ± 0.3^#^	23 ± 0.9
*B. ogadensis*	100	10	13 ± 0.8	12 ± 0.1	9 ± 0.2	8 ± 0.7	17 ± 0.9	17 ± 0.3
	100	20	19 ± 0.8	17 ± 0.3	14 ± 0.2	14 ± 0.2	20 ± 0.6	21 ± 0.9
Positive control	Cefoxitin		S	R	–	–	–	–
	Gentamicin		–	–	R	S	R	S
	Ceftriaxone		–	–	R	S	R	S
Negative control (5% DMSO)			N	N	N	N	N	N

The values represent mean ± standard deviation, *N* NO inhibition zone, *R* Resistant (≤16 mm IZ), *S* Susceptible (≥ 21 mm IZ). Where, ^*^P < 0.05when compared to cefoxitin treated MRSA. While, ^#^*P* < 0.05 when compared to modern drug treated *K. pneumoniae*

Essential oil extracted from *B.cuspidate* had comparably elicited high antibacterial effect than others. It had 22 mm and 25 mm inhibition zone in diameter against MSSA and MRSA respectively. Whereas, MDR *E. coli* and *K. pneumoniae* had 19 mm and 24 mm inhibition zone in diameter at their 20 μl/disc respectively. It had MIC value (1.56 μl/ml) and MBC (3.12 μl/ml) against MRSA and MIC values ranging from 3.12 to 12.5 μl/ml against tested multidrug resistant gram negative bacteria (Table 2).

Essential oil extracted from *T. schimper* had moderate antibacterial effect on tested *Enterobactericeae.* It had 17 mm and 24 mm inhibition zone in diameter against *E. coli* and *K. pneumoniae* respectively (Table 2). It had also appreciable on both MSSA and MRSA (24 mm) inhibition zone in diameter. On the other hand, *B. ogadensis* had 19 mm inhibition zone in diameter and MIC value (3.12 μl/ml) and MBC (6.25 μl/ml) against MRSA. Moreover, it had MIC value ranging 3.12–6.25 μl/ml and MBC 3.12–12.5 μl/ml for tested reference and MDR *E. coli* and *K. pneumoniae*.

### Combined antibacterial effect of essential oils

In our study, the combined essential oils were showed strong inhibitory action against all reference strains and multidrug resistant bacteria at 5 μl/disc (Table 3). Of which, the combined essential oil obtained from *B. cuspidata* and *T. schimper* (1:1 ratio) exhibited the strongest antibacterial activities. It had inhibition zone in diameter 39 mm, 38 mm, 35 mm, and 29 mm on MRSA, MSSA, *K. pneumoniae* (R) and *E.coli* (R) at 20 μl/disc respectively. This mixture had the lowest MIC and MBC values as compared to other mixture. The MIC values for *K. pneumoniae* (R) and MRSA were (0.39 μl/ml) and *E. coli (R)* (1.56 μl/ml). Their MBC values were range from 0.78–3.12 μl/ml for multidrug resistant gram negative bacteria (Table 4). It had more antibacterial effect than modern antibiotics against selected reference and multidrug resistance bacteria (Table 5). Though phytochemical study has not been done on *B. cuspidata*, the presences of the antibacterial compounds presented in both essential oils were synergistic effect between them.

**Table 3:** Inhibition zone (mm) of combined essential oils against MDR bacteria at AAU, 2018

TMP	μl/disc	Gram positive		Gram negative			
		*S.aureus*		*E.coli*		*K. pneumoniae*	
		ATCC	MRSA	ATCC	MDR	ATCC	MDR
*T. schimper + B.cuspidata* (1:1 ratio)	5	19 ± 1.0	18 ± 1.2	20 ± 1.0	21 ± 0.1	27 ± 1.0	27 ± 0.8^#^
	10	29 ± 1.2	29 ± 0.5^**^	23 ± 1.2	25 ± 0.1^*¥*^	29 ± 1.2	28 ± 0.3^#^
	15	33 ± 0.1	33 ± 0.6^**^	28 ± 0.1	26 ± 0.5^*¥*^	33 ± 0.7	34 ± 0.1^##^
	20	38 ± 0.3	39 ± 0.8^***^	29 ± 0.1	29 ± 0.8^*¥¥*^	35 ± 1.4	35 ± 0.5^##^
*B. cuspidata + B. ogadensis* (1:1 ratio)	5	20 ± 0.4	20 ± 0.1	16 ± 0.4	15 ± 0.9	19 ± 0.7	15 ± 0.4
	10	25 ± 0.5	26 ± 0.3^*^	18 ± 0.6	23 ± 0.2	24 ± 0.4	23 ± 0.2
	15	28 ± 0.3	27 ± 0.3^**^	24 ± 0.5	26 ± 0.4^*¥*^	27 ± 1.2	26 ± 0.4^#^
	20	32 ± 0.1	32 ± 0.1^***^	26 ± 0.8	27 ± 0.1^*¥*^	29 ± 0.1	29 ± 0.2^##^
*B. ogadensis + T. schimper* (1:1 ratio)	5	11 ± 0.6	11 ± 0.3	9 ± 0.1	10 ± 0.1	12 ± 0.4	12 ± 0.4
	10	14 ± 0.3	15 ± 0.7	13 ± 0.8	13 ± 0.8	15 ± 0.6	16 ± 0.6
	15	16 ± 0.4	16 ± 0.1	15 ± 0.5	14 ± 0.4	19 ± 0.7	20 ± 0.7
	20	19 ± 0.1	19 ± 0.8	17 ± 0.7	21 ± 0.3	21 ± 0.2	20 ± 0.7
Modern drug		24 ± 0.8	11 ± 1.3(R)	22 ± 0.4	17 ± 0.4(R)	23 ± 0.7	17 ± 0.2(R)

The values represent mean ± standard deviation, *N* NO inhibition zone, *R* Resistant (≤16 mm IZ), S=Susceptible (≥ 21 mm IZ). Where, ^*^P < 0.05, ^**^P < 0.01, ^***^P < 0.001when compared to cefoxitin treated MRSA. While, ^#^*P* < 0.05, ^##^*P* < 0.01when compared to modern drug treated *K. pneumoniae* and ^*¥*^ *P* < 0.05, ^*¥¥*^*P* < 0.01 when compared to modern drug treated *E. coli*

**Table 4:** Inhibitory concentrations of essential oils (μl/ml) against MDR bacteria in MHB at AAU, 2018

TMP	Inhibitory concentration	*S. aurues*		*E.coli*		*K. pneumoniae*	
		ATCC	MRSA	ATCC	MDR	ATCC	MDR
*T*. *schimper*	MIC	3.12	3.12	6.50	6.25	3.12	3.12
	MBC	6.25	6.25	12.5	12.5	3.12	3.12
*B. ogadensis*	MIC	3.12	3.12	6.25	6.25	3.12	3.12
	MBC	6.25	6.25	12.5	12.5	6.25	6.25
*B. cuspidate*	MIC	1.56	1.56	12.5	12.5	3.12	3.12
	MBC	3.12	3.12	25.0	25.0	3.12	3.12
*B. ogadensis + T. schimper*	MIC	3.12	3.12	6.25	6.25	1.56	1.56
	MBC	6.25	6.25	25.0	25.0	1.56	1.56
*T. schimper + B. cuspidata*	MIC	0.39	0.39	1.56	1.56	0.39	0.39
	MBC	0.39	0.39	3.12	3.12	0.78	0.78
*B. cuspidata + B. ogadensis*	MIC	1.56	1.56	6.25	6.25	0.78	0.78
	MBC	1.56	1.56	25.0	25.0	1.56	1.56
ciprofloxacin(5 μg)	MIC	0.15	10.5	0.50	8.00	0.25	9.00
	MBC	0.25	3.00	1.00	4.00	N	N

*N* Not done, *TMP* Traditional medicinal plant, *MHB* Muller Hinton Broth, *MBC M*inimal Bactericidal Concentration, *MIC* Minimal Inhibitory Concentration

**Table 5:** The mean fractional inhibitory concentration index for MDR bacteria at AAU, 2018

Combined	Gram positive MRSA	multidrug resistant gram negative bacteria	
		*E.coli* (R)	*K. pneumoniae* (R)
*B. cuspidata + B.ogadensis*	0.38^b^	0.75^a^	0.25b
*B. cuspidata + B.ogadensis*	1.50^c^	3.00^d^	0.50^b^
*B. ogadensis + T.schimper*	3.00^d^	3.00^d^	1.00^a^

The values represent mean fractional inhibitory concentration index (*n* = 6), where, ^a^X represents partial synergy while ^b^X synergy interaction. ^c^X represents indifference interaction while ^d^X represents antagonistic interaction of essential oil

The combination of *B. cuspidata* and *B. ogadensis* essential oil had 32 mm inhibition zone in diameter against both MSSA and MRSA (Table 3). On the other hand, it showed an effective antibacterial effect on multidrug resistant *K. pneumoniae* and *E. coli.* It had MIC values ranges from 0.78–6.25 μl/ml for tested gram negative and 1.56 μl/ml against MRSA. It had MBC value 1.56 μl/ml for *K.pnemonae* and MRSA and 25.0 μl/ml against multidrug resistant *E.coli.* The combined effects of essential oil obtained from *B.ogadensis* and *T.schimper* had 21 mm and 17 mm inhibition zone in diameter at 20 μl/disc against *K. pneumoniae* (R) and *E. coli* (R) respectively (Table 3). It had also 19 mm inhibition zone in diameter on MSSA and MRSA. The combination of *T. schimper* and *B.ogadensis* had the highest MIC and MBC values for tested gram positive and gram negative bacteria (Table 4). Overall, the combination of each essential oil had more potent antibacterial effect on MRSA and multidrug resistant *K. pneumoniae* and *E. coli* as compare to currently available and affordable antibiotics (Table 1).

The fractional inhibitory concentration index (FIC _index_) revealed that the combined essential oil from *B.cuspidata* and *T.schimper,* and *Blepharis cuspidata* and *Boswellia ogadensis* had synergistic effect MRSA, *E.coli* and *K. pneumoniae* (R). According to the checkerboard method as described elsewhere [24], mixture of essential oil from *B.cuspidata* and *T.schimper* had pronounced synergistic effect on MRSA and *K. pneumoniae* (R). Its FIC index value was 0.375 and 0.25 for MRSA and *K. pneumoniae (R)* respectively. It had strong antibacterial effect on MRSA with 39 mm, 0.39 μl/ml and 0.78 μl/ml inhibition zones in diameter, MIC and MBC values respectively. Likewise, *B. ogadensis* and *B. cuspidata* had highly synergistic interactions against *K. pneumoniae.* Its FIC index was 0.5. It was indifference for *S. aureus* and antagonistic for *E. coli*. Overall, *K. pneumoniae* was most susceptible for combined essential oil. In contrary to this, the combined oil from *B. ogadensis* and *T. schimper* had antagonistic effect for *E. coli (R),* MRSA and *K. pneumoniae* (R) (Table 5).

## Discussion

The multidrug resistant strain of each bacterium has been developing resistant genes or gene products that enable them to resist for tested antibiotic or mechanisms to form multidrug resistant strain. In this case, most of antibiotics treatments administrated by clinicians have been questionable in the majority of our hospitals where there is no drug susceptibility testing facilities. Unless and otherwise, new drugs or modify currently available antibiotics will be revolutionized, it imposes potentially large health and socioeconomic burden on societies and worries future provision of health care services. This finding substantiates previous studies that clinically isolated bacteria showed resistance to the majority of currently available and affordable drugs. This could be developed by either mutations or exchange of genetic information within and between individuals [3]. Another study showed that evolution of antibacterial resistant in human pathogenic and commensal microorganisms due to interaction between antibiotics exposure and horizontally gene(s) transfer by transformation, transduction and conjugation in very dynamic and unpredictable phenomenon [7, 8]. As a result, they resisted for more than two classes of antibiotics and classified as multi drug resistant bacteria [8, 26, 28]. Another studies showed that multidrug resistant bacteria were bearing different resistant mechanisms such as penicillin-binding proteins, drug modification, mutated drug targets, enhanced efflux pump expression and altered membrane permeability. As a result, it has been created a newly emerging and spreading in the bacterial population that compromised the usefulness of all or a majority of drugs [3, 7–10, 24]. Another report showed that resistant traits are not naturally eliminated or reversed. It may be accumulated over time for variety of antibiotics. This can lead to strains with multiple drug resistance, which is more difficult to kill due to reduced treatment option [8]. Those issues have prompted a search for alternative drug (s) from natural bioactive compounds [10, 14, 28].

The medicinal plants that use by healers in Bale zone are promising antibacterial activities and agrees with many researches presented on antimicrobial activities of medicinal plants on multidrug resistant bacteria. This is due to biodiversity coupled with the chemical diversity found within each species [4, 14, 20, 21]. Other studies shown that medicinal plants synthesize and accumulate some secondary metabolites like alkaloid, sterols, terpenes, flavonoids, saponins, glycoside, cyanogenics, tannins, resins, lactones, quinines and volatile oils; compounds that exhibited a broad spectrum [10, 13, 17, 18, 24, 29]. Many previous studies indicated that a number of essential oils contain aldehydes or phenols that used as antimicrobial properties. In many cases, the effective result from the complex interaction between different classes of compounds such as phenols, aldehydes, ketones, alcohols, esters, ethers or hydrocarbons are found in essential oils [15, 28, 30]. Study done by Virender by different solvents of *Euphorbia hirta, Erythrophleum suaveolens* and *Thevetia peruviana* extracts showed antibacterial effect against *ESBL* producing *E.coli, Pseudomonas, Klebsiella,* MRSA*, Salmonella* and *Proteus*. As a consequence, these bioactive compounds derived from medicinal plants used as starting point for synthetic pharmacophores and as industrial raw materials [1, 2, 11, 18]. In this regard, many researchers argued that there are many mechanisms of antimicrobial interaction that produce synergism. Probably the main reasons for this are sequential inhibition of a common biochemical pathway, inhibition of (enzymes, protein synthesis, nucleic acid synthesis), disintegrated the outer membrane [10, 15, 20]. Other authors proposed that, the synergistic effect could be due to the similarity of their mechanism; or may be due to act on the different targets [2, 4, 17].

With other respects, synergistic combination of essential oils of oregano/basil, basil/ bergamot, oregano / bergamot and oregano / perilla against *S. aureus, E. coli, B. subtilis* and *S.cerevisiae* respectively shown that significantly disrupted the integrity of cell membranes [23, 24]. Another study showed that alkaloid in combination with conventional antibiotics (methicillin, ampicillin) exhibited antimicrobial effects against microorganisms [15, 17, 22, 29]. The antimicrobial effect of mixture of the LGEO and amoxicillin indicated synergistic effects against MRSA [23]. However, the commercial turmeric essential oil alone did not show bactericidal effect against the microorganism (*L.monocytogenes* & *S. typhimurium*) but when combined with ascorbic acid, it showed significant antibacterial effect [10, 15, 18, 24].

Inversely, the combined essential oil from *B.ogadensis* and *T. schimper* had antagonistic relations with a FIC index greater than 2 for almost all tested bacteria and resulted less effective in their combination [19, 24]. This result argues with many studies, this might be due to the combination of the essential oils of bactericidal and/or bacteriostatic agents act on the same target of the microorganism and/ or chemical interaction between compounds [18].

A lot has to be done to investigate the undiscovered medicine plants and their valuable chemicals that can potentially curb multidrug resistant bacteria. Essential oils obtained from these medicinal plants have significant potential against MDR bacteria. Their cumulative synergistic effects were inhibited the growth of reference and multidrug resistant bacteria. This interaction may be resulted due to the new structure or reaction or different mechanism of action which lead to easy lethal action of all tested bacteria. The plant may possess therapeutic properties or exert beneficial pharmacological effects on mentioned human pathogen. Yet, their phytochemical composition hasn’t been studied. This is reminding us for searching antibacterial compound and/ or secondary metabolites from plants to overcome the problem from multidrug resistant bacteria.

## Conclusions

Based on the present study combined essential oils were found to have more antibacterial effect than single EO. Even, it is promising anti-bacteria for multi-drug resistant bacteria and the ways to overcome difficulty caused by them. Hence, essential oil contains different bioactive that may be applied to a pharmaceutical composition as modulator or adjuvant or precursor for synthesis new antibiotics in future activities.

## Abbreviations

ATCC: American Type Culture Collection used as reference strain for respective bacteria
CLSI: Clinical Laboratory Standard Institute
DMSO: Dimethyl Sulphoxide
EOs: Essential Oils
ESBL: Extended Spectrum Beta Lactamase
FICI: Fractional Inhibitory Concentration Index
IZ: Inhibition Zone
MBC: Minimal Bactericidal Concentration
MBC: Minimum Bactericidal Concentration
MDR: Multidrug Resistant
MHA: Muller Hinton Agar
MHB: Muller Hinton Broth
MIC: Minimal Inhibitory Concentration
MRSA: Methicillin Resistant *Staphylococcus aureus*
TMP: Traditional Medicine Plant
